# Holographic Properties of Irgacure 784/PMMA Photopolymer Doped with SiO_2_ Nanoparticles

**DOI:** 10.3390/polym15224391

**Published:** 2023-11-13

**Authors:** Jundi Wang, Qingyang Fu, Yaping Zhang, Bing Zhang

**Affiliations:** Yunnan Provincial Key Laboratory of Modern Information Optics, Kunming University of Science and Technology, Kunming 650500, China; 1036885515wilde@gmail.com (J.W.); fuqingyang565@gmail.com (Q.F.)

**Keywords:** silica dioxide nanoparticles, Irgacure 784/PMMA, volume holographic gratings, holographic data storage

## Abstract

To enhance the holographic properties, one of the main methods is increasing the solubility of the photosensitizer and modifying the components to improve the modulation of the refractive index in the photopolymer. This study provides evidence, through the introduction of a mutual diffusion model, that the incorporation of SiO_2_ nanoparticles in photopolymers can effectively enhance the degree of refractive index modulation, consequently achieving the objective of improving the holographic performance of the materials. Different concentrations of SiO_2_ nanoparticles have been introduced into highly soluble photosensitizer Irgacure 784 (solubility up to 10wt%)-doped poly-methyl methacrylate (Irgacure 784/PMMA) photopolymers. Holographic measurement experiments have been performed on the prepared samples, and the experiments have demonstrated that the Irgacure 784/PMMA photopolymer doped with 1.0 $\times \;10^{-3}$wt% SiO_2_ nanoparticles exhibits the highest diffraction efficiency (74.5%), representing an approximate 30% increase in diffraction efficiency as compared to an undoped photopolymer. Finally, we have successfully achieved the recording of real objects on SiO_2_/Irgacure 784/PMMA photopolymers, demonstrated by the SiO_2_/Irgacure 784/PMMA photopolymer material prepared in this study, which exhibits promising characteristics for holographic storage applications. The strategy of doping nanoparticles (Nps) in Irgacure 784/PMMA photopolymers has also provided a new approach for achieving high-capacity holographic storage in the future.

## 1. Introduction

Holographic storage uses the interference principle of light to record information in the form of holograms on storage materials [1,2,3]. The stored information can be retrieved by reconstructing holograms [4]. Photopolymers incorporating poly-methyl methacrylate (PMMA) have emerged as a predominant choice in the burgeoning field of holographic storage, which is a focal point of contemporary research, attributed to their affordability, minimal shrinkage, ample storage capacity, adjustable thickness, and heightened stability and security [5,6,7,8,9]. The information storage capacity of photopolymers is closely related to their holographic properties. Incorporating nanoparticles with varying refractive indices from the monomer has been proven to effectively enhance the refractive index modulation, thus improving the holographic properties of photopolymers [10,11,12].

The purpose of doping nanoparticles into photopolymer materials is to enhance the refractive index modulation of the material by using high-refractive-index nanoparticles as the refractive index modulation component [13]. This increases the refractive index difference between the bright area (filled with photoproducts) and the dark area (filled with nanoparticles) during the exposure process, thereby achieving the goal of improving the holographic performance of the material [14]. This technique, pursued by numerous researchers over the years, has seen the exploration and utilization of various nanoparticles to this end, as delineated in several key studies [15,16,17,18,19,20,21]. In 2008, Luo et al. [18] produced a material with a diffraction efficiency of 49.3% by doping a 1.6 mm thick PQ/PMMA photopolymer material with SiO_2_ nanoparticles. In 2013, Lee et al. [19] introduced gold nanoparticles into a PQ/PMMA photopolymer with a thickness of 1.5 mm. The localized surface plasmon resonance (LSPR) effect of the gold nanoparticles was utilized to enhance the photopolymerization effect, resulting in increased grating strength and a doubled diffraction efficiency (from 23.3% to 47.1%). Moreover, the shrinkage rate of the material was only 0.17%. In 2019, Zhu et al. [20] introduced graphene oxide (GO) into PQ/PMMA photopolymers and confirmed the phase modulation capability of GO photopolymers. They successfully recorded a fork grating on the material and reconstructed a vortex beam. In 2022, Hu et al. [21] incorporated fullerene into a PQ/PMMA system, increasing the intensity diffraction efficiency of the material to 72%.

While numerous nanoparticle doping strategies have facilitated advancements in PQ/PMMA photopolymer materials, the potential of these materials remains constrained due to the limited solubility of the photosensitizer PQ [22,23]. Specifically, its solubility in MMA stands at a mere 0.7wt%, thereby establishing an upper threshold for the efficacy of PQ/PMMA photopolymer materials [24]. As a crucial component of the photochemical reaction of photopolymer materials, increased solubility of the photosensitizer PQ in the material directly correlates with enhanced performance of the photopolymer material, which is a well-established observation. Subsequent studies have been conducted to improve the solubility of the photosensitizer PQ by adding solvents such as BZMA [25] and THFMA [26] (up to 1.3wt% solubility), but the results were not substantial. In recent years, researchers have focused on a highly soluble photosensitizer, Irgacure 784, TI. Compared to the photosensitizer PQ, Irgacure 784 can reach a maximum solubility of 10wt% in MMA [27]. It has been demonstrated that Irgacure 784/PMMA photopolymer materials perform better than PQ/PMMA photopolymer materials in terms of sensitivity, response time, and diffraction efficiency under the same experimental conditions [28,29,30]. It is apparent that Irgacure 784 holds promise as the favored photosensitizer for a new generation of photopolymer materials. Building upon existing research on the influence of doping with nanoparticles on photopolymer materials, we anticipate the diffraction efficiency of photopolymer materials will be improved by introducing nanoparticle components that amplify the refractive index modulation to the Irgacure 784/PMMA system. However, research in this area is currently rather limited. Therefore, we aim to provide new research directions and improve the holographic performance of photopolymer materials through our study.

Doping nanoparticles proves to be an effective technique in enhancing the refractive index modulation of photopolymers and is widely used in the preparation of photopolymers. Numerous studies cited above demonstrate the effectiveness of this approach in enhancing the holographic properties of photopolymers. When the photopolymer is undoped with nanoparticles, the combination of Irgacure 784 + PMMA already has good holographic properties. Unfortunately, research on nanoparticle doping in this system is severely restricted. The purpose of this investigation is to explore the holographic storage potential of the 784 PMMA system with SiO_2_ nanoparticles experimentally. The reasons for using SiO_2_ nanoparticles are that they have excellent physical properties such as high hardness and high transparency, and they do not participate in the photochemical reaction of the material during the exposure process. In this study, we establish a mutual diffusion model for the photopolymer material system and conduct numerical simulations to analyze the impact of the addition of nanoparticle components on the refractive index modulation of the material. On this basis, SiO_2_ nanoparticles of varying concentrations have been dispersed in Irgacure 784/PMMA photopolymer material. By measuring the diffraction efficiency of Irgacure 784/PMMA photopolymers doped with different concentrations of SiO_2_ nanoparticles, it has been demonstrated that the doping of SiO_2_ nanoparticles can enhance the diffraction efficiency of photopolymer materials. In this study, the material doped with 1.0 × 10^−3^wt% SiO_2_ nanoparticles exhibits the highest diffraction efficiency, which is almost doubled as compared to that of the undoped material. Finally, we perform holographic recording of real objects on the photopolymer material doped with 1.0 × 10^−3^wt% SiO_2_ nanoparticles and obtain clear and stable holographic reconstructed images. This proves that the high-performance holographic photopolymer material developed in this study has the potential to meet the demand for large-capacity volume holographic recording. It also provides a new approach to explore methods for improving the performance of holographic storage materials in the future.

## 2. Mutual Diffusion Model

Introducing nanoparticles into photopolymer materials as doping agents leverages their ability to serve as refractive index modulating elements within the substrate. Nanoparticles occupying the dark region induce more photosensitizers to diffuse from the dark to the light region, resulting in a higher consumption of photosensitizers in the light region. This achieves the goal of increasing the photoproduct yield. (The photoproducts are the result of the photoinitiator undergoing a polymerization reaction with the material substrate under the influence of light. The generation of photoproducts is the primary cause of the refractive index grating formation and constitutes the main factor influencing the degree of refractive index modulation in the material [22]). During the exposure process, the photosensitizer Irgacure 784 is changed into excited molecules by light and photopolymerized with the monomer MMA in the bright regions to form the photoproducts. As the consumption rate of the photosensitizer molecules in the bright regions is faster than that in the dark regions, the corresponding concentration gradient is generated between the bright and dark regions. The photosensitizer molecules in the dark regions diffuse to the bright regions due to their concentration difference, which increases the chemical potential energy in the bright region. Subsequently, the nanoparticles in the bright regions are affected by the high chemical potential energy used to diffuse from the bright regions to the dark regions, resulting in an increase in the concentration of nanoparticles in the dark regions. Due to the mutual diffusion effect between the monomer and the nanoparticles, the concentration of the photosensitizer in the bright regions of the material is higher after full exposure, resulting in more photoproducts. While in the dark regions, a large number of nanoparticles with large refractive index differences from the monomer are distributed, forming a steady refractive index modulation. At the completion of exposure, the photoproducts and unconsumed photosensitizer molecules are mostly concentrated in the bright regions, while the dark regions are filled by a large number of nanoparticles [14,31,32].

To investigate the impact of introducing silicon dioxide nanoparticles on photopolymer materials, we employ the photopolymer material mutual diffusion model [33,34,35,36] to analyze the physical mechanisms of spatial transfer between different components within the material:

(1) $$\left\{\begin{array}{c} \frac{\partial C_{\left[TI\right]}\left(x,t\right)}{\partial t}=D\left\{C_{\left[SiO_{2}\right]}\left(x,t\right)\frac{\partial^{2}C_{\left[TI\right]}\left(x,t\right)}{\partial x^{2}}-C_{\left[TI\right]}\left(x,t\right)\frac{\partial^{2}C_{\left[SiO_{2}\right]}\left(x,t\right)}{\partial x^{2}}\right\}-F\left(x,t\right)C_{\left[TI\right]}\left(x,t\right)\\ \frac{\partial C_{\left[SiO_{2}\right]}\left(x,t\right)}{\partial t}=D\left\{C_{\left[TI\right]}\left(x,t\right)\frac{\partial^{2}C_{\left[SiO_{2}\right]}\left(x,t\right)}{\partial x^{2}}-C_{\left[SiO_{2}\right]}\left(x,t\right)\frac{\partial^{2}C_{\left[TI\right]}\left(x,t\right)}{\partial x^{2}}\right\}\frac{\partial C_{\left[P\right]}\left(x,t\right)}{\partial t}\\ \frac{\partial C_{\left[P\right]}\left(x,t\right)}{\partial t}=\int_{0}^{t}F\left(x,t\right)C_{\left[TI\right]}\left(x,t\right) \end{array}\right.$$

In Equation (1), the photosensitizer used is Irgacure 784 (TI), and the dopant is $SiO_{2}$ nanoparticles, with the photoproduct (P) as the output. $C_{\left[.\right]}\left(x,t\right)$ is the spatiotemporal distribution of the concentration of the corresponding component, i.e., TI, $SiO_{2}$, or P. *D* is the diffusion coefficient of system, and $F\left(x,t\right)$ is the spatiotemporal distribution of polymerization rate.

(2) $$F\left(x,t\right)=k\cdot I\left(x,t\right)$$

In Equation (2), k=Φεd, where Φ is the quantum yield, ε is the molar absorption coefficient, and d is the thickness of the material. Finally, $I\left(x,t\right)$ is the distribution of the incident light field.

To investigate the impact of introducing SiO_2_ nanoparticles onto the photopolymer material, we conducted numerical simulations by substituting the following initial conditions into Equation (1). In this paper, the transmission grating recorded by photopolymer material is used. The angle between the two incident angles is 25°. The incident light field is expressed as $I\left(x,t\right)=I_{0}\left[1+V\cos\left(K_{g}x\right)\right]$ [37]. Since the irradiation intensity $I\left(x,t\right)$ used in the article is constant, $I\left(x,t\right)$ is a constant function of time. Similarly, the polymerization rate $F\left(x,t\right)$ is a constant function that does not change with time. This means that the generation rate of photoproducts mentioned in the article is constant. Because all $C_{\left[.\right]}\left(x,t\right)$ in Equation (1) are functions of time t, the addition of t variables to I and F simply correspond to $C_{\left[.\right]}\left(x,t\right)$.

**Figure 1:**
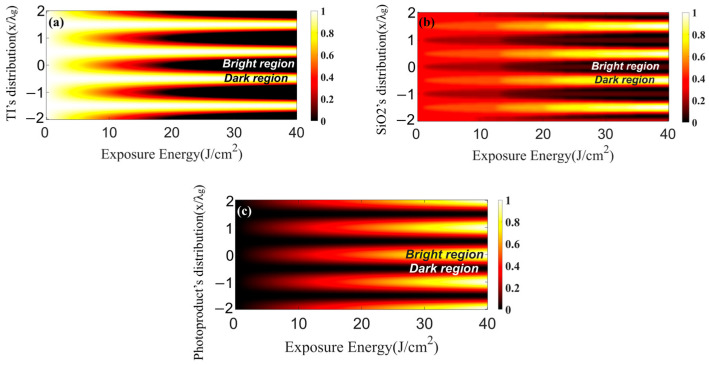
Spatial distribution of different components under continuous exposure: (**a**) photosensitizer TI; (**b**) SiO_2_ nanoparticles; (**c**) photoproducts.

The exposing fringe visibility V is unity, and the grating constant is $K_{g}=2\pi ∕\lambda_{g}$, with the grating period being $\lambda_{g}=1.03\;\mu m$. The initial light intensity is set to 5 mW/cm^2^. The initial concentration conditions of each component are set as $C_{\left[TI\right]}\left(x,0\right)=1.2\times 10^{-4}\;mol∕cm^{3}$ and $C_{\left[SiO_{2}\right]}\left(x,0\right)=3\times 10^{-8}\;mol∕cm^{3}$; the diffusion coefficient is $D=3.2\times 10^{-8}\;cm^{2}∕s$. This paper mainly analyzes the influence of introducing SiO_2_ nanoparticles onto the whole material by comparing the spatial distribution of TI molecule concentration in the system of doped SiO_2_ nanoparticles and undoped SiO_2_ nanoparticles under the same conditions. The concentration of TI, photoproducts, and SiO_2_ in photopolymers varies with time t. However, since the accumulated exposure energy over time is the direct cause of this change, the coordinate axis below (a, b, c) in Figure 1 is J/cm^2^, which describes the energy. Figure 1 depicts the spatial distribution of the component concentrations ($C_{\left[.\right]}\left(x,t\right)$) within the photopolymer system as a function of exposure energy, with the exposure energy being directly proportional to the exposure time.

Figure 1a–c depicts the spatial distribution of the various components during the exposure process. Figure 1 displays varying concentrations through a color gradient, ranging from light to dark shades. The lighter hues indicate higher concentrations, while the darker hues indicate lower concentrations.

As exposure time increases, the concentration of the photosensitizer TI in the bright regions decreases (appearing in dark color); correspondingly, the concentration of photoproducts generated by the photo-induced polymerization reaction in the bright regions increases with time. The photosensitizer TI in the dark regions is influenced by the concentration gradient and thus flows towards the bright regions, driving the SiO_2_ nanoparticles in the bright regions to diffuse in the opposite direction into the dark regions. After the grating reaches a steady state, the photoproducts in the bright regions and the nanoparticles in the dark regions form a steady refractive index modulation.

**Figure 2:**
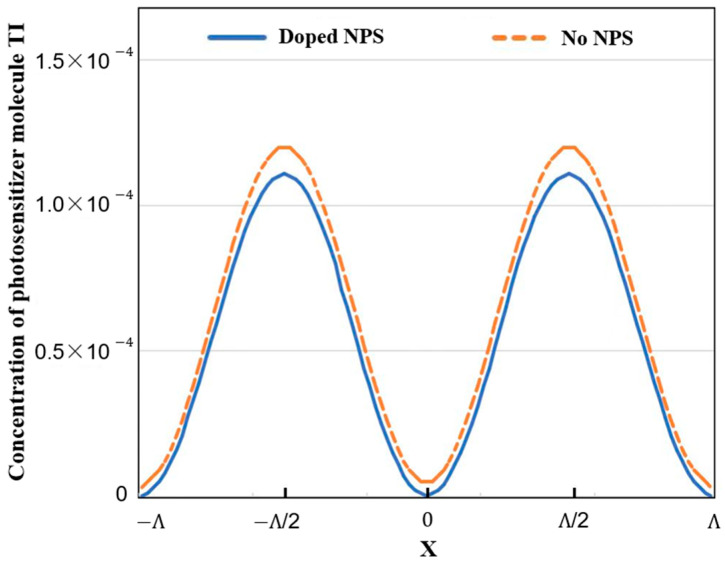
Photosensitizer TI in photopolymer doped and undoped SiO_2_ nanoparticles at t = 2500 s.

Figure 2 illustrates the spatial distribution of the photosensitizer (TI) in the photopolymer materials with and without nanoparticle doping at the end of the exposure, while keeping the kinetic parameters used in the simulation constant. It is readily apparent that the introduction of SiO_2_ nanoparticles significantly enhances the consumption of the photosensitizer TI molecules within the illuminated region. This effect indicates a higher diffusion rate of photosensitizer TI molecules from the dark regions to the illuminated regions, resulting in a further increase in the quantity of photoproducts within the bright areas. 

As a result, this facilitates the targeted augmentation of refractive index modulation, consequently elevating the holographic performance of the material.

## 3. Preparation Process

The components of photopolymer materials are shown in Table 1.

**Figure 3:**
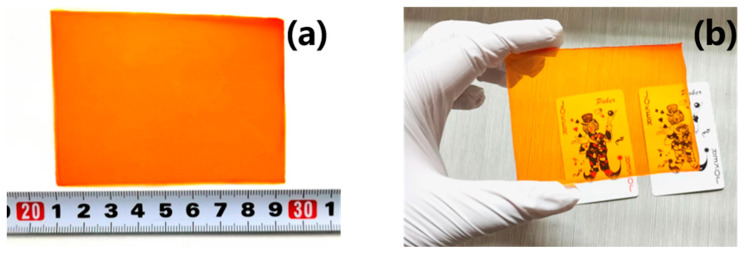
SiO_2_/Irgacure 784/PMMA photopolymer material finished product. (**a**) Prepared photopolymer, (**b**) “Jokers” under photopolymer.

To make a SiO_2_/Irgacure 784/PMMA photopolymer sample, we adopted the optimized thermal polymerization method [38]. SiO_2_ nanoparticles were dispersed in methyl isobutyl ketone (MIBK) solution and shaken in an ultrasonic water bath for 3–5 h. The well-dispersed SiO_2_/MIBK solution and MMA monomer were added to the reagent bottle. Then, Irgacure 784 photosensitizer (4wt%) and 2,2-azobisisobutyronitrile (AIBN) thermal initiator (1.2wt%) were dissolved in a mixed solution and ultrasonically shaken in a water bath at 60 °C for 20 min to ensure uniform mixing. The compositions of the mixture were kept as MMA:AIBN:TI = 100:1.2:4. The reagent bottle was then continuously stirred on a magnetic stirrer and kept at a constant temperature of 60 °C until the solution was viscous. The mixture was poured into a glass mold with a thickness of 1.5 mm and placed horizontally in an oven at 60 °C for 48 h to be solidified. The finished product of the experiment is shown in Figure 3. The material prepared using this process is suitable for holographic storage. 

## 4. Material Characteristics

### 4.1. Spectra Measurements

**Figure 4:**
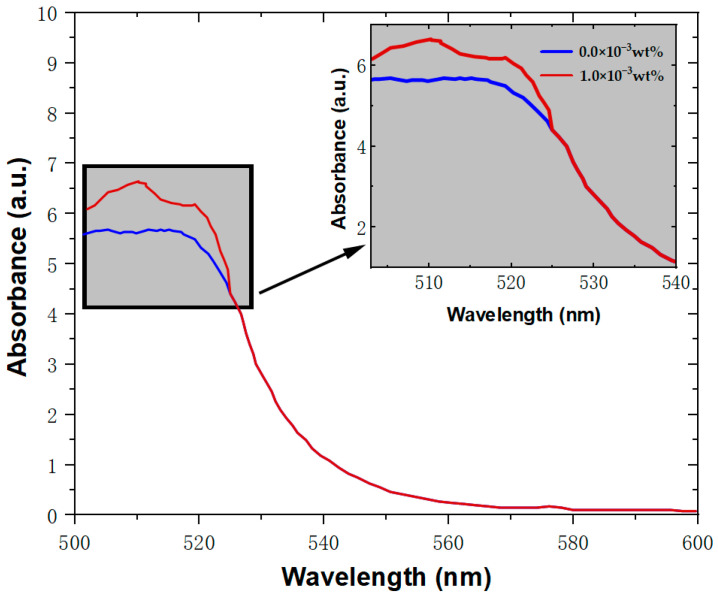
Absorption spectra of photopolymer materials doped with 1.0 $\times \;10^{-3}$wt% SiO_2_ nanoparticles and undoped nanoparticles.

The photosensitivity of photopolymers determines the holographic properties of the materials. The materials prepared in this research were analyzed utilizing a TU-1901 dual-beam UV–V spectrophotometer. The absorption spectra of SiO_2_ nanoparticle materials and undoped nanoparticle materials at the same photosensitizer concentration (4wt%) were obtained, as shown in Figure 4.

Figure 4 reveals that incorporating SiO₂ nanoparticles has a negligible impact on the material’s absorption coefficient within the 530 nm–550 nm wavelength range. However, at an approximately 514 nm band, there is a significant surge in the absorption coefficient, denoting high susceptibility to laser interactions in this region. The utilization of lasers operating near this wavelength is likely to induce substantial holographic scattering and consequent material loss, indicating its unsuitability for holographic recording endeavors. Therefore, a 532 nm green DPSS laser was selected as the recording light for the material.

### 4.2. Diffraction Efficiency

**Figure 5:**
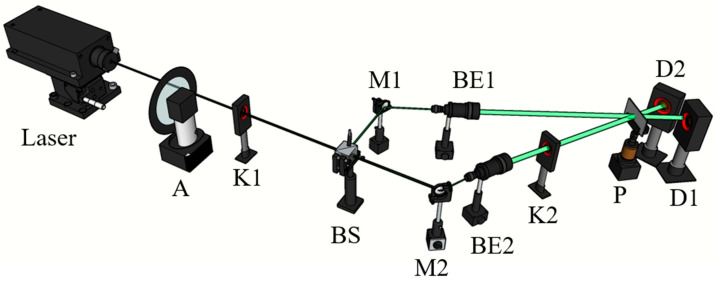
Experimental setup for holographic recording by green laser (532 nm). M1, M2: Mirror; A: Attenuator; BS: Beam splitter; BE1, BE2: Beam expander; K1, K2: Shutter; D1, D2: Laser power meter probe; P: Photopolymer.

To measure the optical properties of the material, we built the optical system as shown in Figure 5. A laser beam of 532 nm was split into two equal power laser beams to incident on the material, and the angle between the two beams was 22°. Laser power of 5 mW was used to irradiate the photopolymer for recording. The experimental results show that the interference spot of the two beams irradiated the sample and gave a circular spot with a radius of about 2 cm.

During the measurement of diffraction efficiency, the first shutter (K1) remained open while the second shutter (K2) was opened for 49 s and then closed for 1 s. This process was repeated 20 times. The light intensity was recorded through laser power meter probe 1 (D1) and laser power meter probe 2 (D2) for the transmitted ($I_{0}$) and diffracted light ($I_{+1}$) intensities, respectively. These recorded values were then used to calculate the diffraction efficiency of the material. The material is thick (1.5 mm) and has a certain absorption and reflection of the recording light (532 nm). If we ignore the absorption and the Fresnel reflection, the diffraction efficiency (η) can be defined as follows:

(3) $$\eta =\frac{I_{+1}}{I_{0}+I_{+1}}\;$$

where $I_{0}$ and $I_{+1}$ are the intensities of the transmitted and first-order diffracted beams. The recording sensitivity (S) of the material can be expressed as follows [21]:

(4) $$S=\frac{1}{Id}(\frac{\partial \sqrt{\eta }}{\partial t})$$

where I here represents the intensity of recording wave, d is photopolymer material thickness, and η is the diffraction efficiency of the material.

In this study, five groups of experiments (A1~A7) were designed and compared based on the different concentrations of doped nanoparticles. The mass fractions of each component among the groups are shown in Table 2.

**Figure 6:**
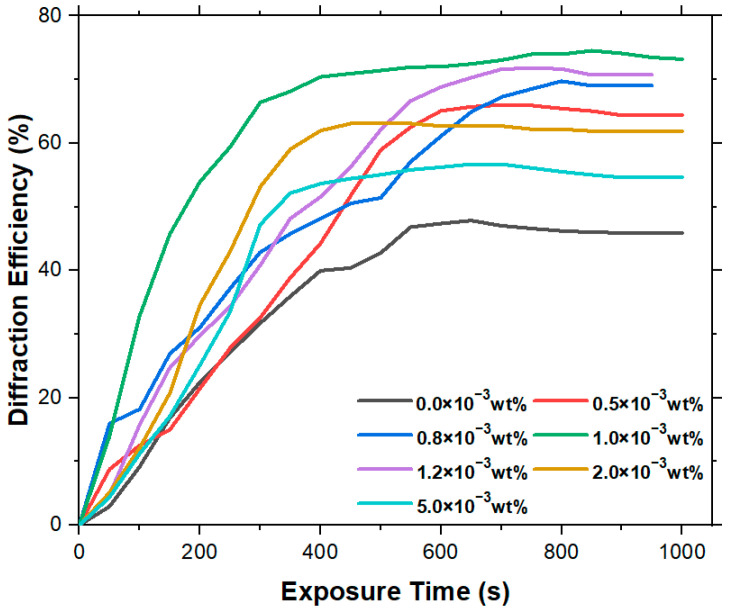
Fitting curve of diffraction efficiency vs. exposure time for materials containing 0.0 $\times \;10^{-3}$wt%, 0.5 $\times \;10^{-3}$wt%, 1.0 $\times \;10^{-3}$wt%, 2.0 $\times \;10^{-3}$wt%, and 5.0 $\times \;10^{-3}$wt% SiO_2_ nanoparticles.

For SiO_2_/Irgacure 784/PMMA photopolymers doped with SiO_2_ nanoparticles of different concentrations (0.0 $\times \;10^{-3}$wt%, 0.5 $\times \;10^{-3}$wt%, 0.8 $\times \;10^{-3}wt\%$, 1.0 $\times \;10^{-3}$wt%, 1.2 $\times \;10^{-3}wt\%$, 2.0 $\times \;10^{-3}$wt%, and 5.0 $\times \;10^{-3}$wt%), the different diffraction efficiency curves are shown in Figure 6. 

**Figure 7:**
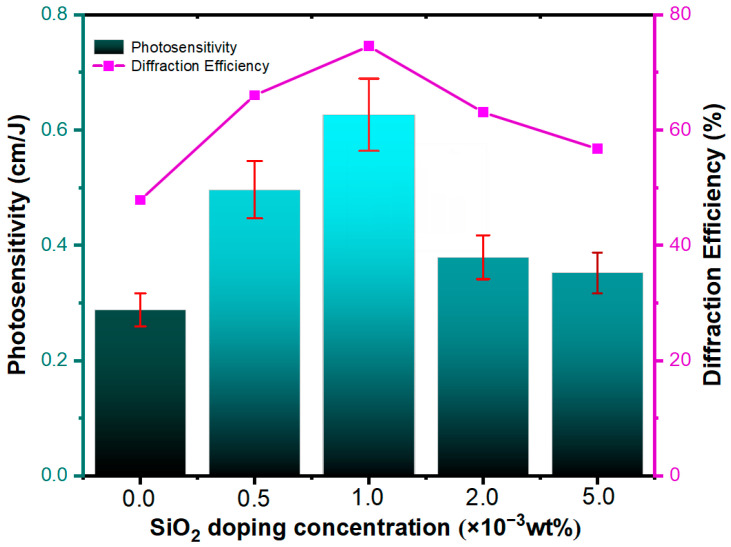
SiO_2_ doping concentration-dependent intensity holographic diffraction efficiency (Magenta line) and photosensitivity (Cyan column).

From Figure 6, it is apparent that the introduction of nanoparticles at various concentrations induces varying degrees of enhancement in the diffraction efficiency of Irgacure 784/PMMA photopolymer materials. Group A4 (doped with 1.0 $\times \;10^{-3}$wt% SiO_2_ nanoparticles) exhibits the highest diffraction efficiency (74%), with a time to reach saturation diffraction efficiency of 850 s. Group A6 (doped with 2.0 $\times \;10^{-3}$wt% SiO_2_ nanoparticles) attains saturation diffraction efficiency fastest (at 450 s), with a maximum diffraction efficiency of 63%. Through an analytical comparison of the experimental data presented in this study with findings from other researchers (as delineated in Table 3), it is unequivocally apparent that there is a positive effect of doping SiO_2_ nanoparticles on the diffraction efficiency of Irgacure 784/PMMA photopolymer materials. Figure 7 presents data on the maximum diffraction efficiency and sensitivity of photopolymers doped with varying concentrations of SiO_2_ nanoparticles. The doping of SiO_2_ nanoparticles improves the diffraction efficiency of the materials but also leads to a significant increase in the sensitivity of the photopolymer materials. Of the materials observed, the photopolymer material doped with 1.0 $\times \;10^{-3}$wt% of SiO_2_ nanoparticles has a sensitivity that is increased by about 2.25 times compared to the Irgacure 784/PMMA photopolymer. Through an analytical comparison of the experimental data presented in this study with findings from other researchers (as delineated in Table 3), it is unequivocally apparent that the positive effect of doping SiO_2_ nanoparticles on the maximum diffraction efficiency and sensitivity of Irgacure 784/PMMA photopolymer materials. Thus, the introduction of SiO_2_ nanoparticles of varying concentrations as a new component can improve the holographic properties of the material.

### 4.3. Recording and Reconstruction of Object Volume Hologram

**Figure 8:**
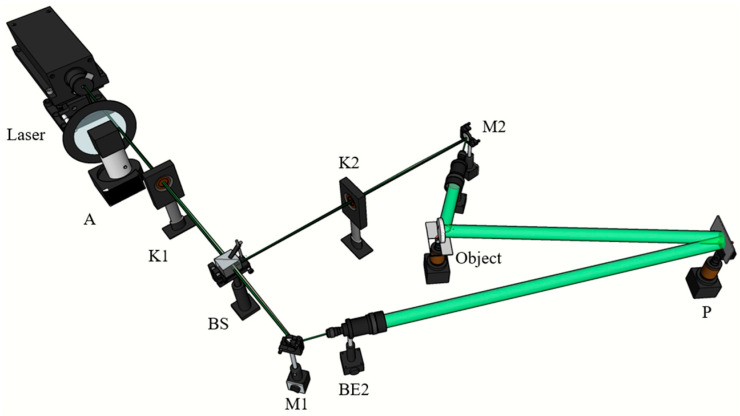
Volume holographic recording. A: Attenuator; K1, K2: Shutter; BS: Beam splitter; BE2: Beam expander; M1, M2: Mirror; P: Photopolymer.

To authenticate the capacity of the materials to record volume holograms, the experimental arrangement used is depicted in Figure 8.

A volume hologram of an object was taken, as shown in Figure 8, and the A2 group of the sample with the highest diffraction efficiency was selected for the measurement. The sample was placed at a designated position, and the shutters K1 and K2 were opened so that the reference light and the object light could illuminate the material at the same time. When the set time was reached, both K1 and K2 were closed and the hologram recording was completed.

**Figure 9:**
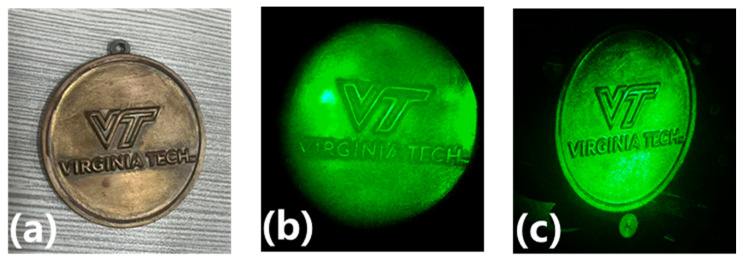
Volume holographic recording and reconstruction of real objects. (**a**) Coin, (**b**) reconstruction of the coin using Irgacure 784/PMMA photopolymer, (**c**) reconstruction of the coin using SiO_2_/Irgacure 784/PMMA photopolymer.

Then, K1 was opened and K2 was closed for the hologram reconstruction; the original object and the reconstruction of the hologram are shown in Figure 9.

Figure 9 presents the holographic reconstructed images of the undoped nanoparticle photopolymer material and the nanoparticle-doped photopolymer material. It is evident that the clarity of the holographic reconstructed images has been significantly improved with the SiO_2_/Irgacure 784/PMMA photopolymer.

## 5. Conclusions

SiO_2_ nanoparticles with different concentrations were doped into Irgacure 784/PMMA photopolymer, and a 1.5 mm thick SiO_2_/Irgacure 784/PMMA photopolymer was prepared. By employing a mutual diffusion model, the concentration changes in different components within the photopolymer material during the exposure process were numerically simulated. This study provides evidence that the introduction of SiO_2_ nanoparticles enables the formation of a steady-state refractive index modulation within the photopolymer. Furthermore, during the diffusion process, the presence of SiO_2_ nanoparticles induces the influx of a greater number of photosensitizer molecules into the illuminated region, leading to increased consumption and subsequent generation of photoproducts. Ultimately, this approach facilitates the anticipated increase in the degree of refractive index modulation in the photopolymer. Subsequently, optical property measurements were performed on photopolymer materials doped with nanoparticles of varying concentrations. It was found that the material doped with 1.0 $\times \;10^{-3}$wt% SiO_2_ nanoparticles exhibits the highest diffraction efficiency, which has been greatly improved compared to the material without nanoparticles (from 47.8% to 74.5%). The photopolymer material doped with 1.0 $\times \;10^{-3}$wt% SiO_2_ nanoparticles was utilized for holographic recording and reconstruction, exhibiting definitive and stable recording outcomes. The results of our experiments indicate that holographic properties are improved when the Irgacure 784/PMMA photopolymer is doped with SiO_2_ nanoparticles. Further investigation is recommended to determine the effectiveness of doping with other nanoparticles, and this study can be used as a reference for enhancing holographic properties using the polymer system studied. The synthesized material demonstrates excellent holographic properties and great potential in the application of holographic recording and storage.

## Figures and Tables

**Table 1:** Main components of photopolymer materials.

Name	Chemical Formula	Appearance	Purity (%)
MMA	C_5_H_8_O_2_	Colorless liquid	99.5
AIBN	C_8_H_12_N_4_	Crystalline powder	99.0
Irgacure 784	C_30_H_22_F_4_N_2_Ti	Orange powder	99.2
MIBK	C_6_H_12_O	Colorless liquid	99.5
Quartz	SiO_2_	White powder (particle diameter: 20 nm)	99.0

**Table 2:** Experimental grouping of doped SiO_2_ nanoparticles with different concentrations.

Components	MMA	Irgacure 784	AIBN	Quartz
A1 (wt%)	100.0	4.0	1.2	$0.0\times 10^{-3}$
A2 (wt%)	100.0	4.0	1.2	$0.5\times 10^{-3}$
A3 (wt%)	100.0	4.0	1.2	$0.8\times 10^{-3}$
A4 (wt%)	100.0	4.0	1.2	$1.0\times 10^{-3}$
A5 (wt%)	100.0	4.0	1.2	$1.2\times 10^{-3}$
A6 (wt%)	100.0	4.0	1.2	$2.0\times 10^{-3}$
A7 (wt%)	100.0	4.0	1.2	$5.0\times 10^{-3}$

**Table 3:** Comparison of diffraction efficiency and sensitivity of Irgacure 784/PMMA photopolymer materials with different ratios.

Materials	Nanoparticles	S (cm/J)	$\eta_{sat}$(%)	Year/Ref.
PQ (1.0wt%)/PMMA	-	0.027	3.3	2017 [29]
PQ (1.2wt%)/PMMA	SiO_2_	-	49.3	2011 [34]
PQ (1.2wt%)/PMMA	C60	0.59	72.0	2022 [21]
Irgacure 784 (5.0wt%)/PMMA	-	0.571	52.0	2017 [29]
Irgacure 784 (4.0wt%)/PMMA	-	0.288	47.8	This study
Irgacure 784 (4.0wt%)/PMMA	SiO_2_	0.627	74.5	This study

## Data Availability

Data are contained within the article.
